# Supplementary material for: Climate and Bedrock Collectively Influence the Diversity Pattern of Plant Communities in Qiniangshan Mountain
Source: Plants (Basel). 2024 Dec 20;13(24):3567. doi: 10.3390/plants13243567 (PMC11677607; doi:10.3390/plants13243567)
Supplement: Supplementary file 1 [file plants-13-03567-s001.zip › Supplementary materials-plants-2024.12.20.pdf]

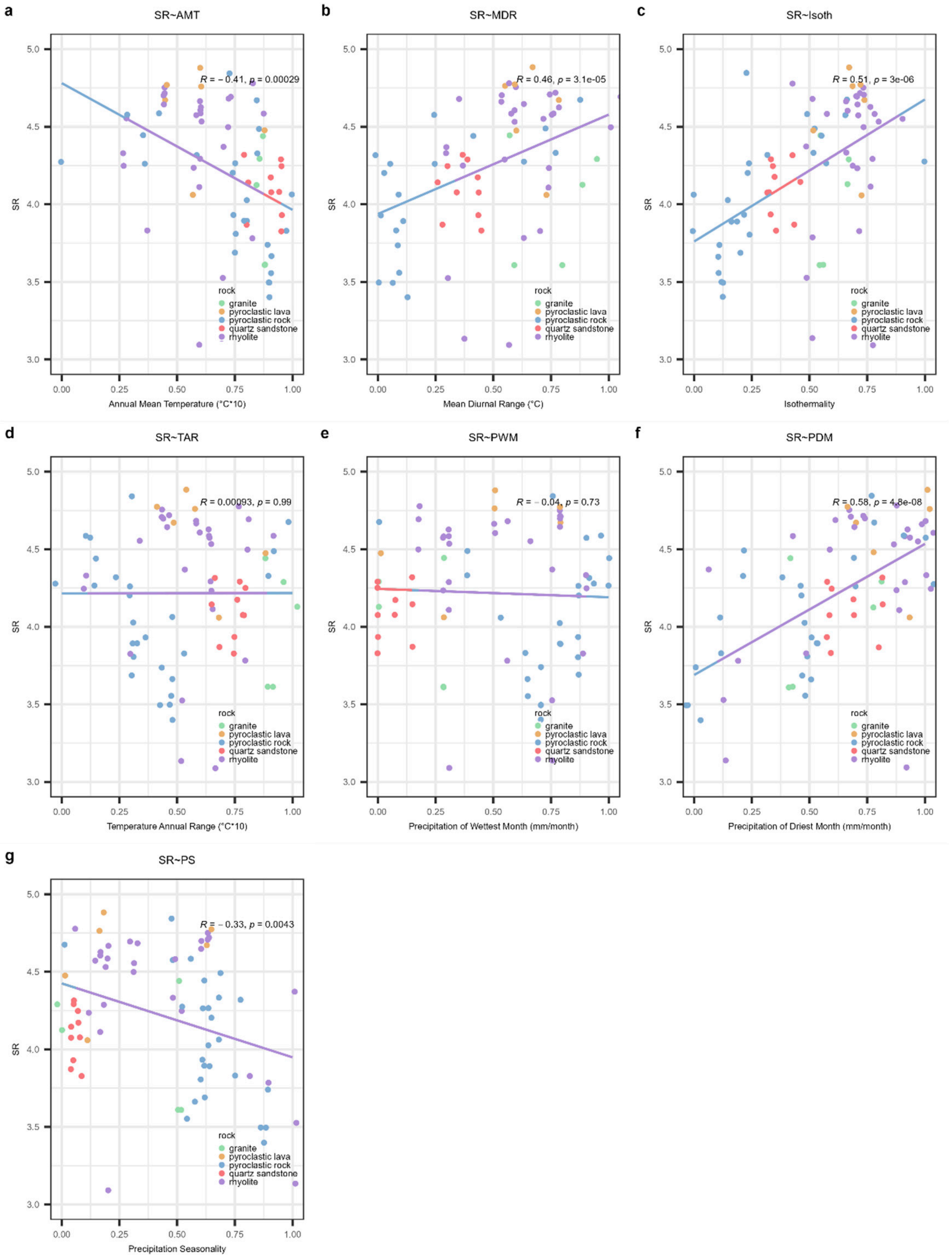

Figure S1 Interaction effects between climate variables on species richness (SR) in Qiniangshan Mountain. **a, g** AMT and PS were negatively correlated with the species richness of plant communities in Qiniangshan Mountain. **b, c, f** MDR, Isoth and PDM were positively correlated with the species richness of plant communities in Qiniangshan Mountain. **d, e** TAR and PWM were no significant correlation with the species richness of plant communities in Qiniangshan Mountain.

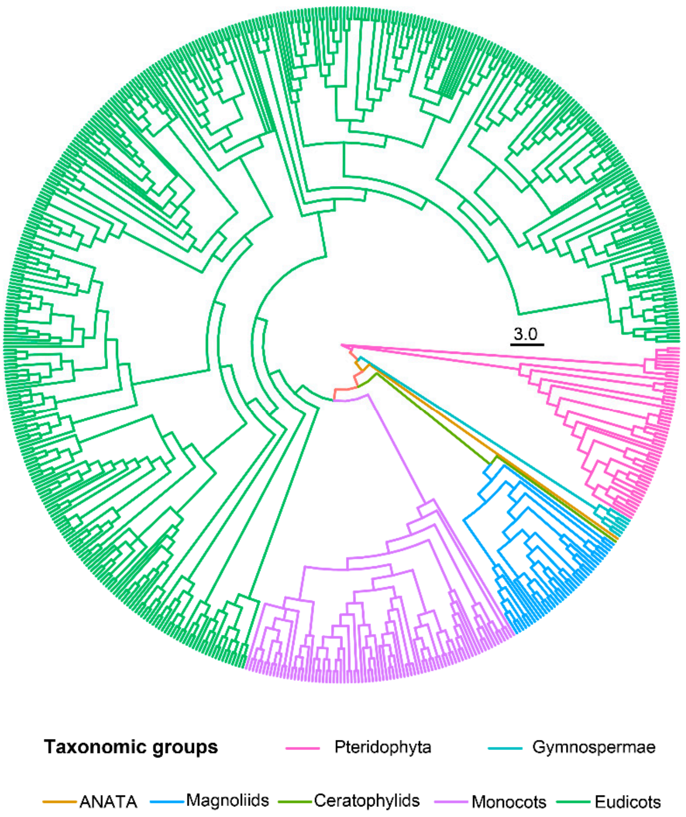

Figure S2 Vascular plants phylogenetic tree of plant communities in Qiniangshan Mountain.

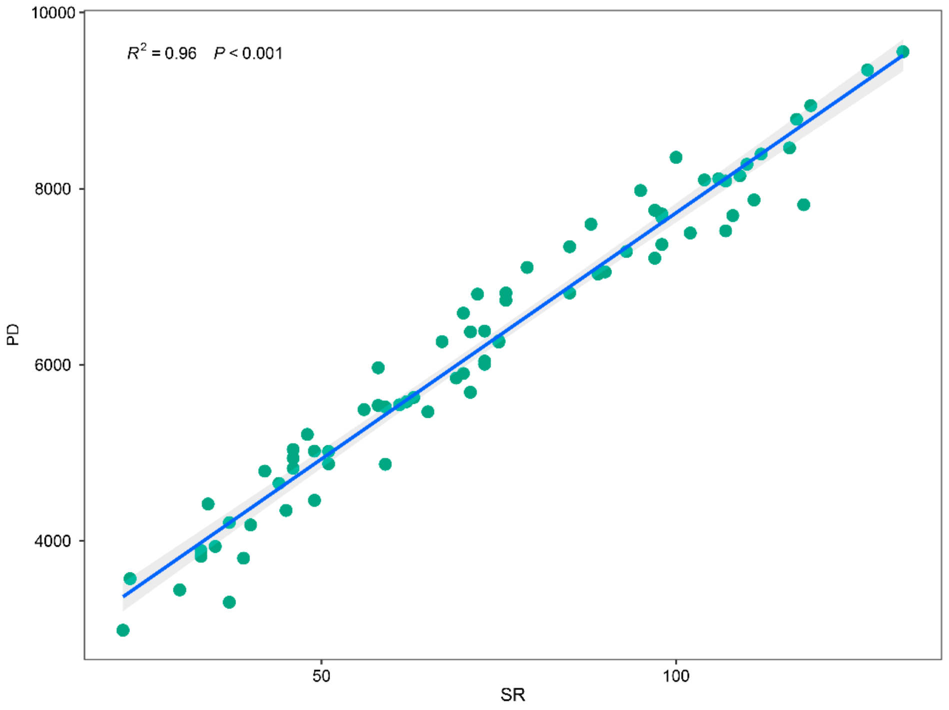

Figure S3 Linear regression results of phylogenetic diversity and species richness of plant communities in Qiniangshan Mountain.

Table S1 Basic information of plant community quadrats in Qiniangshan Mountain

Quadrat number	Longitude	Latitude	Elevation/m	Area/m^2	Bedrock type
BZ-1	114°31'41.36"	22°32'25.60"	68	1200	granite
DBSC-1	114°32'14.12"	22°31'38.38"	440	1200	rhyolite
DBSC-2	114°32'16.11"	22°31'39.84"	433	1200	pyroclastic lava
GL-1	114°31'53.41"	22°32'33.63"	20	1200	rhyolite
GL-2	114°31'58.02"	22°32'37.50"	218	1200	rhyolite
GL-6	114°32'16.86"	22°32'50.37"	272	1200	rhyolite
GL-7	114°32'15.42"	22°32'47.08"	263	1200	rhyolite
GL-8	114°32'17.25"	22°32'49.57"	271	1200	rhyolite
GL-11	114°32'58.39"	22°32'25.63"	350	1200	rhyolite
GL-15	114°32'11.39"	22°33'6.47"	133	1200	pyroclastic rock
GL-19	114°32'42.82"	22°32'58.95"	234	1200	rhyolite
GPX-1	114°34'49.18"	22°30'55.29"	515	1200	pyroclastic rock
GPX-2	114°34'54.44"	22°31'3.82"	664	1200	pyroclastic rock
GPX-3	114°34'55.08"	22°30'46.14"	462	1200	pyroclastic rock
GPX-4	114°35'18.25"	22°30'27.69"	152	1200	pyroclastic rock
GPX-5	114°35'33.42"	22°30'17.24"	203	1200	pyroclastic rock
GPX-6	114°35'43.31"	22°30'12.45"	160	1200	pyroclastic rock
GPX-7	114°35'58.30"	22°29'51.07"	60	1200	pyroclastic rock
LBA-1	114°35'44.05"	22°30'31.59"	197	1200	pyroclastic rock
LZ-4	114°35'8.32"	22°32'21.21"	56	1200	quartz sandstone
LZ-7	114°36'17.68"	22°32'23.84"	87	1200	quartz sandstone
LZ-10	114°35'59.54"	22°32'9.67"	99	1200	quartz sandstone
LZ-11	114°35'58.88"	22°32'23.63"	20	1200	quartz sandstone
LZ-12	114°35'40.09"	22°32'20.08"	24	1200	quartz sandstone
MLH-5	114°36'48.42"	22°30'37.56"	145	1200	pyroclastic rock
XD-1	114°31'41.13"	22°31'57.82"	65	1200	pyroclastic rock
XHG-5	114°31'57.54"	22°30'56.13"	199	1200	rhyolite
XHG-6	114°31'48.90"	22°31'55.74"	78	1200	pyroclastic rock
XHG-7	114°31'55.60"	22°32'0.74"	126	1200	rhyolite
XSH-1	114°33'26.38"	22°32'13.21"	205	1200	pyroclastic lava
XSH-2	114°33'19.99"	22°32'7.88"	283	1200	pyroclastic lava
YMK-1	114°33'43.61"	22°32'33.40"	109	1200	pyroclastic lava
YMK-2	114°33'26.69"	22°32'25.27"	172	1200	rhyolite
YMK-3	114°33'11.75"	22°32'22.76"	280	1200	rhyolite
YMK-11	114°34'0.88"	22°32'1.11"	145	1200	rhyolite
YMK-12	114°34'23.83"	22°31'39.07"	140	1200	rhyolite
ZF-25	114°32'26.89"	22°31'31.04"	720	1200	rhyolite
ZF-26	114°32'32.98"	22°31'29.53"	815	1200	pyroclastic rock
ZF-27	114°32'21.51"	22°31'31.72"	646	1200	rhyolite
ZF-28	114°32'17.97"	22°31'33.71"	573	1200	pyroclastic lava
ZF-29	114°32'9.23"	22°31'37.64"	443	1200	rhyolite
ZF-30	114°32'5.13"	22°31'40.92"	331	1200	rhyolite

**Quadrat number**	**Longitude**	**Latitude**	**Elevation/m**	**Area/m^2**	**Bedrock type**
BZ-2	114°31'44.86"	22°32'28.89"	120	400	granite
BZ-3	114°31'39.67"	22°32'25.32"	55	400	granite
DC-2	114°35'22.61"	22°29'44.36"	73	400	pyroclastic rock
DC-4	114°35'27.75"	22°29'42.74"	59	400	pyroclastic rock
DC-5	114°35'20.63"	22°29'50.61"	132	400	pyroclastic rock
DC-6	114°35'19.36"	22°29'52.86"	158	400	pyroclastic rock
GL-9	114°32'48.57"	22°32'46.89"	225	400	rhyolite
GL-10	114°32'56.87"	22°32'33.85"	337	400	pyroclastic lava
GL-12	114°32'0.81"	22°32'36.37"	231	400	rhyolite
GL-13	114°32'6.40"	22°32'40.66"	260	400	rhyolite
GL-14	114°32'9.90"	22°32'40.98"	280	400	rhyolite
GL-16	114°32'5.58"	22°33'8.35"	247	400	granite
GL-17	114°32'5.59"	22°33'8.13"	268	400	granite
GPX-8	114°36'8.93"	22°29'57.66"	66	400	pyroclastic rock
GPX-9	114°36'18.34"	22°29'59.90"	67	400	pyroclastic rock
GPX-10	114°35'40.27"	22°30'21.78"	177	400	pyroclastic rock
GPX-11	114°35'33.07"	22°30'19.26"	96	400	pyroclastic rock
LBA-2	114°35'56.44"	22°30'25.24"	81	400	pyroclastic rock
LBA-3	114°36'3.12"	22°30'10.75"	137	400	pyroclastic rock
LZ-1	114°35'14.60"	22°32'24.73"	104	400	quartz sandstone
LZ-3	114°35'13.95"	22°32'27.96"	55	400	quartz sandstone
LZ-6	114°36'17.38"	22°32'24.59"	110	400	quartz sandstone
LZ-8	114°36'5.41"	22°32'1.54"	57	400	quartz sandstone
LZ-9	114°36'5.57"	22°32'1.54"	28	400	quartz sandstone
MLH-1	114°36'39.90"	22°30'9.33"	24	400	pyroclastic rock
MLH-2	114°36'41.46"	22°30'9.81"	33	400	pyroclastic rock
MLH-3	114°36'21.15"	22°30'0.63"	60	400	pyroclastic rock
XHG-2	114°31'51.08"	22°31'2.07"	149	400	rhyolite
XHG-4	114°31'56.87"	22°30'55.65"	195	400	rhyolite
XHG-8	114°31'58.97"	22°30'56.65"	250	400	rhyolite
XHG-9	114°32'2.44"	22°30'59.55"	280	400	rhyolite
YMK-14	114°34'30.50"	22°31'9.16"	351	400	rhyolite
YMK-15	114°34'32.70"	22°31'6.49"	390	400	rhyolite

Table S2 Climate factors of community quadrats preserved based on collinearity analysis

**Climatic factors**	**Plant community quadrats**
BIO1 = Annual Mean Temperature [° C*10]	√
BIO2 = Mean Diurnal Range [° C]	√
BIO3 = Isothermality	√
BIO4 = Temperature Seasonality [standard deviation]	
BIO5 = Max Temperature of Warmest Month [° C*10]	
BIO6 = Min Temperature of Coldest Month [° C*10]	
BIO7 = Temperature Annual Range [° C*10]	√
BIO8 = Mean Temperature of Wettest Quarter [° C*10]	
BIO9 = Mean Temperature of Driest Quarter [° C*10]	
BIO10 = Mean Temperature of Warmest Quarter [° C*10]	
BIO11 = Mean Temperature of Coldest Quarter [° C*10]	
BIO12=Annual Precipitation [mm/year]	
BIO13 = Precipitation of Wettest Month [mm/month]	√
BIO14 = Precipitation of Driest Month [mm/month]	√
BIO15 = Precipitation Seasonality [coefficient of variation]	√
BIO16 = Precipitation of Wettest Quarter [mm/quarter]	
BIO17 = Precipitation of Driest Quarter [mm/quarter]	
BIO18 = Precipitation of Warmest Quarter [mm/quarter]	
BIO19 = Precipitation of Coldest Quarter [mm/quarter]	

Table S3 GLM and SAR analysis of species richness, phylogenetic structure and environmental factors of plant communities in Qiniangshan Mountain

<table>
<tr><td colspan="9">SR of plant communities</td></tr>
<tr><td rowspan="2">predictor variables</td><td colspan="4">GLM analysis</td><td colspan="4">SAR analysis</td></tr>
<tr><td>Beta_Hat</td><td>SE</td><td>t</td><td>Pr(>|t|)</td><td>Beta_Hat</td><td>SE</td><td>z</td><td>Pr(>|z|)</td></tr>
<tr><td>Intercept</td><td>2.3689</td><td>0.3701</td><td>6.401</td><td>1.85e-08***</td><td>0.72663</td><td>0.37306</td><td>1.9478</td><td>0.0514435</td></tr>
<tr><td>pyroclastic lava</td><td>0.4231</td><td>0.2016</td><td>2.099</td><td>0.039642*</td><td>0.23964</td><td>0.12879</td><td>1.8607</td><td>0.0627916</td></tr>
<tr><td>pyroclastic rock</td><td>0.4419</td><td>0.1962</td><td>2.253</td><td>0.027599*</td><td>0.35887</td><td>0.14624</td><td>2.4540</td><td>0.0141267</td></tr>
<tr><td>quartz sandstone</td><td>0.5323</td><td>0.5323</td><td>2.405</td><td>0.018982*</td><td>0.40206</td><td>0.13654</td><td>2.9447</td><td>0.0032325</td></tr>
<tr><td>rhyolite</td><td>0.3153</td><td>0.1686</td><td>1.870</td><td>0.065869</td><td>0.19832</td><td>0.10925</td><td>1.8153</td><td>0.0694805</td></tr>
<tr><td>MDR</td><td>1.6051</td><td>0.4074</td><td>3.940</td><td>0.000199***</td><td>1.16489</td><td>0.30461</td><td>3.8242</td><td>0.0001312</td></tr>
<tr><td>Isoth</td><td>-1.5102</td><td>0.5762</td><td>-2.621</td><td>0.010865*</td><td>-1.11352</td><td>0.36683</td><td>-3.0355</td><td>0.0024011</td></tr>
<tr><td>PDM</td><td>1.6848</td><td>0.3854</td><td>4.372</td><td>4.47e-05***</td><td>1.08213</td><td>0.27489</td><td>3.9366</td><td>8.266e-05</td></tr>
<tr><td>PS</td><td>1.0870</td><td>0.3894</td><td>2.792</td><td>0.006852**</td><td>0.73736</td><td>0.22062</td><td>3.3422</td><td>0.0008311</td></tr>
<tr><td></td><td>R^2 = 0.520</td><td colspan="3">AIC: 50.106</td><td>R^2 = 0.686</td><td colspan="3">AIC: 44.345, (AIC for lm: 50.106)</td></tr>
<tr><td></td><td colspan="4">Moran's I = -0.062531</td><td colspan="4">Moran's I = -0.02544</td></tr>
<tr><td colspan="9">NRI of plant communities</td></tr>
<tr><td rowspan="2">predictor variables</td><td colspan="4">GLM analysis</td><td colspan="4">SAR analysis</td></tr>
<tr><td>Beta_Hat</td><td>SE</td><td>t</td><td>Pr(>|t|)</td><td>Beta_Hat</td><td>SE</td><td>z</td><td>Pr(>|z|)</td></tr>
<tr><td>Intercept</td><td>-2.3981</td><td>0.9826</td><td>-2.441</td><td>0.01720*</td><td>-2.37199</td><td>0.98887</td><td>-2.3987</td><td>0.0164535</td></tr>
<tr><td>aspect</td><td>0.8299</td><td>0.3384</td><td>2.452</td><td>0.01669*</td><td>0.85053</td><td>0.33539</td><td>2.5360</td><td>0.0112139</td></tr>
<tr><td>TAR</td><td>3.4067</td><td>1.1337</td><td>3.005</td><td>0.00368**</td><td>3.39242</td><td>1.27414</td><td>2.6625</td><td>0.0077557</td></tr>
<tr><td>PWM</td><td>3.8454</td><td>1.2010</td><td>3.202</td><td>0.00206**</td><td>3.81119</td><td>1.38664</td><td>2.7485</td><td>0.0059868</td></tr>
<tr><td>PS</td><td>-3.5555</td><td>0.6567</td><td>-5.414</td><td>8.17e-07***</td><td>-3.56705</td><td>1.06381</td><td>-3.3531</td><td>0.0007991</td></tr>
<tr><td></td><td>R^2 = 0.391</td><td colspan="3">AIC: 177.24</td><td>R^2 = 0.392</td><td colspan="3">AIC: 181.12, (AIC for lm: 177.24)</td></tr>
<tr><td></td><td colspan="4">Moran's I = -0.022921</td><td colspan="4">Moran's I = -0.0012094</td></tr>
<tr><td colspan="9">NTI of plant communities</td></tr>
<tr><td rowspan="2">predictor variables</td><td colspan="4">GLM analysis</td><td colspan="4">SAR analysis</td></tr>
<tr><td>Beta_Hat</td><td>SE</td><td>t</td><td>Pr(>|t|)</td><td>Beta_Hat</td><td>SE</td><td>z</td><td>Pr(>|z|)</td></tr>
<tr><td>Intercept</td><td>-4.3139</td><td>2.0301</td><td>-2.125</td><td>0.037335*</td><td>-2.815817</td><td>1.191382</td><td>-2.3635</td><td>0.018104</td></tr>
<tr><td>pyroclastic lava</td><td>-0.5559</td><td>0.5755</td><td>-0.966</td><td>0.337599</td><td>-0.771469</td><td>0.373486</td><td>-2.0656</td><td>0.038867</td></tr>
<tr><td>pyroclastic rock</td><td>0.1914</td><td>0.5956</td><td>0.321</td><td>0.748990</td><td>-0.053020</td><td>0.404252</td><td>-0.1312</td><td>0.895652</td></tr>
<tr><td>quartz sandstone</td><td>0.3076</td><td>0.6590</td><td>0.467</td><td>0.642245</td><td>0.085143</td><td>0.351805</td><td>0.2420</td><td>0.808766</td></tr>
<tr><td>rhyolite</td><td>-0.3190</td><td>0.4802</td><td>-0.664</td><td>0.508864</td><td>-0.296839</td><td>0.301695</td><td>-0.9839</td><td>0.325164</td></tr>
<tr><td>Isoth</td><td>-0.9259</td><td>0.8151</td><td>-1.136</td><td>0.260136</td><td>-0.570484</td><td>0.487949</td><td>-1.1691</td><td>0.242345</td></tr>
<tr><td>TAR</td><td>4.3345</td><td>1.6596</td><td>2.612</td><td>0.011139*</td><td>2.746005</td><td>1.013788</td><td>2.7087</td><td>0.006756</td></tr>
<tr><td>PWM</td><td>3.0130</td><td>1.3340</td><td>2.259</td><td>0.027218*</td><td>2.070603</td><td>0.777261</td><td>2.6640</td><td>0.007722</td></tr>
<tr><td>PDM</td><td>2.4899</td><td>0.6690</td><td>3.722</td><td>0.000411***</td><td>1.463935</td><td>0.444988</td><td>3.2898</td><td>0.001002</td></tr>
<tr><td></td><td>R^2 = 0.203</td><td colspan="3">AIC: 205.97</td><td>R^2 = 0.486</td><td colspan="3">AIC: 202.16, (AIC for lm: 205.97)</td></tr>
<tr><td></td><td colspan="4">Moran's I = 0.12223</td><td colspan="4">Moran's I = -0.036438</td></tr>
<tr><td colspan="9">PDI of plant communities</td></tr>
<tr><td rowspan="2">predictor variables</td><td colspan="4">GLM analysis</td><td colspan="4">SAR analysis</td></tr>
<tr><td>Beta_Hat</td><td>SE</td><td>t</td><td>Pr(>|t|)</td><td>Beta_Hat</td><td>SE</td><td>z</td><td>Pr(>|z|)</td></tr>
</table>

Intercept	3.4204	1.3708	2.495	0.014949*	3.6921	1.6428	2.2474	0.024615
Isoth	1.3059	0.6793	1.922	0.058622	1.3739	1.0291	1.3350	0.181883
TAR	-3.7999	1.3340	-2.849	0.005762**	-4.3160	1.4076	-3.0661	0.002168
PWM	-2.4045	1.0054	-2.391	0.019472*	-2.7043	1.2029	-2.2481	0.024570
PDM	-2.5103	0.6521	-3.850	0.000259***	-2.6340	0.8172	-3.2232	0.001268
	**R^2 = 0.216**	**AIC: 205.13**			**R^2 = 0.376**	**AIC: 201.18, (AIC for lm: 205.13)**		
	**Moran's I = 0.23865**				**Moran's I = -0.0065203**			

Table S4 List of vascular plants of plant community quadrats in Qiniangshan Mountain

Taxonomic groups	Family	Species
Pteridophyta	Lycopodiaceae	*Lycopodium japonicum* Thunb.
Pteridophyta	Lycopodiaceae	*Palhinhaea cernua* (L.) Vasc. & Franco
Pteridophyta	Selaginellaceae	*Selaginella biformis* A. Braun ex Kuhn
Pteridophyta	Selaginellaceae	*Selaginella delicatula* (Desv.) Alston
Pteridophyta	Selaginellaceae	*Selaginella doederleinii* Hieron.
Pteridophyta	Selaginellaceae	*Selaginella involvens* (Sw.) Spring
Pteridophyta	Selaginellaceae	*Selaginella tamariscina* (P. Beauv.) Spring
Pteridophyta	Selaginellaceae	*Selaginella trachyphylla* Hieron.
Pteridophyta	Selaginellaceae	*Selaginella uncinata* (Desv.) Spring
Pteridophyta	Osmundaceae	*Plenasium vachellii* (Hook.) C. Presl
Pteridophyta	Gleicheniaceae	*Dicranopteris linearis* (Burm. f.) Underw.
Pteridophyta	Gleicheniaceae	*Dicranopteris pedata* (Houtt.) Nakaike
Pteridophyta	Gleicheniaceae	*Diplopterygium chinense* (Rosenst.) De Vol
Pteridophyta	Gleicheniaceae	*Diplopterygium glaucum* (Thunb. ex Houtt.) Nakai
Pteridophyta	Lygodiaceae	*Lygodium flexuosum* (L.) Sw.
Pteridophyta	Lygodiaceae	*Lygodium japonicum* (Thunb.) Sw.
Pteridophyta	Lygodiaceae	*Lygodium microphyllum* (Cav.) R. Br.
Pteridophyta	Cibotiaceae	*Cibotium barometz* (L.) J. Sm.
Pteridophyta	Lindsaeaceae	*Lindsaea chienii* Ching
Pteridophyta	Lindsaeaceae	*Lindsaea cultrata* (Willd.) Sw.
Pteridophyta	Lindsaeaceae	*Lindsaea ensifolia* Sw.
Pteridophyta	Lindsaeaceae	*Lindsaea heterophylla* Dryand.
Pteridophyta	Lindsaeaceae	*Lindsaea orbiculata* (Lam.) Mett. ex Kuhn
Pteridophyta	Lindsaeaceae	*Odontosoria chinensis* J. Sm.
Pteridophyta	Pteridaceae	*Adiantum capillus-veneris* L.
Pteridophyta	Pteridaceae	*Adiantum flabellulatum* L.
Pteridophyta	Pteridaceae	*Pteris cretica* L.
Pteridophyta	Pteridaceae	*Pteris ensiformis* Burm.
Pteridophyta	Pteridaceae	*Pteris semipinnata* L.
Pteridophyta	Dennstaedtiaceae	*Dennstaedtia scabra* (Wall.) Moore
Pteridophyta	Aspleniaceae	*Asplenium yoshinagae* Makino
Pteridophyta	Blechnaceae	*Blechnopsis orientalis* (L.) C. Presl
Pteridophyta	Blechnaceae	*Woodwardia harlandii* Hook.
Pteridophyta	Blechnaceae	*Woodwardia japonica* (L. f.) Sm.
Pteridophyta	Thelypteridaceae	*Cyclosorus parasiticus* (L.) Farw.
Pteridophyta	Thelypteridaceae	*Pronephrium simplex* (Hook.) Holttum
Pteridophyta	Thelypteridaceae	*Stegnogramma sagittifolia* (Ching) L. J. He & X. C. Zhang
Pteridophyta	Dryopteridaceae	*Bolbitis subcordata* (Copel.) Ching
Pteridophyta	Dryopteridaceae	*Dryopteris fuscipes* C. Chr.
Pteridophyta	Dryopteridaceae	*Dryopteris podophylla* (Hook.) Kuntze
Pteridophyta	Dryopteridaceae	*Dryopteris sieboldii* (van Houtte ex Mett.) Kuntze
Pteridophyta	Dryopteridaceae	*Dryopteris sparsa* (Buch.-Ham. ex D. Don) Kuntze

Taxonomic groups	Family	Species
Pteridophyta	Dryopteridaceae	*Polystichum balansae* Christ
Pteridophyta	Nephrolepidaceae	*Nephrolepis cordifolia* (L.) C. Presl
Pteridophyta	Polypodiaceae	*Drynaria coronans* J. Sm.
Pteridophyta	Polypodiaceae	*Lemmaphyllum diversum* (Rosenst.) De Vol & C. M. Kuo
Pteridophyta	Polypodiaceae	*Lemmaphyllum microphyllum* C. Presl
Pteridophyta	Polypodiaceae	*Lepisorus ovatus* (Wall. ex Bedd.) C. F. Zhao, R. Wei & X. C. Zhang
Pteridophyta	Polypodiaceae	*Lepisorus thunbergianus* (Kaulf.) Ching
Pteridophyta	Polypodiaceae	*Pyrrosia adnascens* (Sw.) Ching
Pteridophyta	Polypodiaceae	*Pyrrosia lingua* (Thunb.) Farw.
Gymnospermae	Gnetaceae	*Gnetum luofuense* C. Y. Cheng
Gymnospermae	Gnetaceae	*Gnetum montanum* Markgr.
Gymnospermae	Gnetaceae	*Gnetum parvifolium* (Warb.) C. Y. Cheng ex Chun
Gymnospermae	Pinaceae	*Pinus massoniana* Lamb.
Gymnospermae	Podocarpaceae	*Podocarpus macrophyllus* (Thunb.) Sweet
Gymnospermae	Taxaceae	*Amentotaxus argotaenia* (Hance) Pilg.
Angiospermae	Schisandraceae	*Kadsura heteroclita* (Roxb.) Craib
Angiospermae	Piperaceae	*Piper hongkongense* C. DC.
Angiospermae	Aristolochiaceae	*Asarum insigne* Diels
Angiospermae	Magnoliaceae	*Michelia skinneriana* Dunn
Angiospermae	Annonaceae	*Artabotrys hongkongensis* Hance
Angiospermae	Annonaceae	*Desmos chinensis* Lour.
Angiospermae	Annonaceae	*Fissistigma glaucescens* (Hance) Merr.
Angiospermae	Annonaceae	*Fissistigma oldhamii* (Hemsl.) Merr.
Angiospermae	Annonaceae	*Uvaria boniana* Finet & Gagnep.
Angiospermae	Annonaceae	*Uvaria grandiflora* Roxb.
Angiospermae	Annonaceae	*Uvaria macrophylla* Roxb.
Angiospermae	Lauraceae	*Beilschmiedia fordii* Dunn
Angiospermae	Lauraceae	*Beilschmiedia tsangii* Merr.
Angiospermae	Lauraceae	*Cassytha filiformis* L.
Angiospermae	Lauraceae	*Cinnamomum appelianum* Schewe
Angiospermae	Lauraceae	*Cinnamomum camphora* (L.) J. Presl
Angiospermae	Lauraceae	*Cinnamomum jensenianum* Hand.-Mazz.
Angiospermae	Lauraceae	*Cinnamomum parthenoxylon* (Jack) Meisn.
Angiospermae	Lauraceae	*Cinnamomum subavenium* Miq.
Angiospermae	Lauraceae	*Cinnamomum validinerve* Hance
Angiospermae	Lauraceae	*Cinnamomum wilsonii* Gamble
Angiospermae	Lauraceae	*Cryptocarya chinensis* (Hance) Hemsl.
Angiospermae	Lauraceae	*Cryptocarya concinna* Hance
Angiospermae	Lauraceae	*Lindera aggregata* (Sims) Kosterm.
Angiospermae	Lauraceae	*Lindera communis* Hemsl
Angiospermae	Lauraceae	*Lindera nacusua* (D. Don) Merr.
Angiospermae	Lauraceae	*Litsea acutivena* Hayata
Angiospermae	Lauraceae	*Litsea cubeba* (Lour.) Pers.

Taxonomic groups	Family	Species
Angiospermae	Lauraceae	*Litsea glutinosa* (Lour.) C. B. Rob.
Angiospermae	Lauraceae	*Litsea kwangtungensis* Hung T. Chang
Angiospermae	Lauraceae	*Litsea rotundifolia* var. *oblongifolia* (Nees) C. K. Allen
Angiospermae	Lauraceae	*Machilus breviflora* (Benth.) Hemsl.
Angiospermae	Lauraceae	*Machilus chekiangensis* S. K. Lee
Angiospermae	Lauraceae	*Machilus chinensis* (Champ. ex Benth.) Hemsl.
Angiospermae	Lauraceae	*Machilus thunbergii* Siebold & Zucc.
Angiospermae	Lauraceae	*Machilus velutina* Champ. ex Benth.
Angiospermae	Lauraceae	*Neolitsea aurata* (Hayata) Koidz.
Angiospermae	Lauraceae	*Neolitsea cambodiana* Lecomte
Angiospermae	Lauraceae	*Neolitsea phanerophlebia* Merr.
Angiospermae	Chloranthaceae	*Sarcandra glabra* (Thunb.) Nakai
Angiospermae	Acoraceae	*Acorus gramineus* Soland.
Angiospermae	Araceae	*Amorphophallus konjac* K. Koch
Angiospermae	Araceae	*Pothos chinensis* (Raf.) Merr.
Angiospermae	Dioscoreaceae	*Dioscorea cirrhosa* Lour.
Angiospermae	Dioscoreaceae	*Dioscorea fordii* Prain & Burkill
Angiospermae	Dioscoreaceae	*Dioscorea linearicordata* Prain & Burkill
Angiospermae	Triuridaceae	*Sciaphila ramosa* Fukuy. & T. Suzuki
Angiospermae	Pandanaceae	*Pandanus austrosinensis* T. L. Wu
Angiospermae	Pandanaceae	*Pandanus kaida* Kurz.
Angiospermae	Pandanaceae	*Pandanus tectorius* Parkinson
Angiospermae	Melanthiaceae	*Veratrum schindleri* Loes.
Angiospermae	Smilacaceae	*Smilax china* L.
Angiospermae	Smilacaceae	*Smilax davidiana* A. DC.
Angiospermae	Smilacaceae	*Smilax glabra* Roxb.
Angiospermae	Smilacaceae	*Smilax glaucochina* Warb.
Angiospermae	Smilacaceae	*Smilax hypoglauca* Benth.
Angiospermae	Smilacaceae	*Smilax lanceifolia* Roxb.
Angiospermae	Orchidaceae	*Ania hongkongensis* (Rolfe) Tang & F. T. Wang
Angiospermae	Orchidaceae	*Bulbophyllum ambrosia* (Hance) Schltr.
Angiospermae	Orchidaceae	*Bulbophyllum kwangtungense* Schltr.
Angiospermae	Orchidaceae	*Cionisaccus procera* (Ker Gawl.) M. C. Pace
Angiospermae	Orchidaceae	*Cleisostoma simondii* var. *guangdongense* Z. H. Tsi
Angiospermae	Orchidaceae	*Coelogyne fimbriata* Lindl.
Angiospermae	Orchidaceae	*Cymbidium sinense* (Jack. ex Andr.) Willd.
Angiospermae	Orchidaceae	*Liparis bootanensis* Griff.
Angiospermae	Orchidaceae	*Liparis campylostalix* Rchb. f.
Angiospermae	Orchidaceae	*Liparis nervosa* (Thunb. ex A. Murray) Lindl.
Angiospermae	Orchidaceae	*Neuwiedia zollingeri* var. *singapureana* (Baker) de Vogel
Angiospermae	Orchidaceae	*Pholidota chinensis* Lindl.
Angiospermae	Orchidaceae	*Tainia dunnii* Rolfe
Angiospermae	Asphodelaceae	*Dianella ensifolia* (L.) DC.

Taxonomic groups	Family	Species
Angiospermae	Asparagaceae	*Asparagus cochinchinensis* (Lour.) Merr.
Angiospermae	Asparagaceae	*Aspidistra lurida* Ker Gawl.
Angiospermae	Asparagaceae	*Aspidistra minutiflora* Stapf
Angiospermae	Asparagaceae	*Liriope spicata* (Thunb.) Lour.
Angiospermae	Asparagaceae	*Ophiopogon bodinieri* H. Lév.
Angiospermae	Arecaceae	*Calamus rhabdocladus* Burret
Angiospermae	Arecaceae	*Calamus tetradactylus* Hance
Angiospermae	Arecaceae	*Calamus thysanolepis* Hance
Angiospermae	Arecaceae	*Phoenix loureiroi* Kunth
Angiospermae	Arecaceae	*Rhapis excelsa* (Thunb.) A. Henry
Angiospermae	Commelinaceae	*Floscopa scandens* Lour.
Angiospermae	Commelinaceae	*Pollia japonica* Thunb.
Angiospermae	Zingiberaceae	*Alpinia hainanensis* K. Schum. in Engler
Angiospermae	Zingiberaceae	*Alpinia japonica* (Thunb.) Miq.
Angiospermae	Zingiberaceae	*Alpinia oblongifolia* Hayata
Angiospermae	Zingiberaceae	*Alpinia stachyodes* Hance
Angiospermae	Zingiberaceae	*Alpinia zerumbet* (Pers.) B. L. Burtt & R. M. Sm.
Angiospermae	Cyperaceae	*Carex adrienii* E. G. Camus in Lecomte
Angiospermae	Cyperaceae	*Carex brunnea* Thunb. in Murray
Angiospermae	Cyperaceae	*Carex chinensis* Retz.
Angiospermae	Cyperaceae	*Carex cruciata* Wahlenb.
Angiospermae	Cyperaceae	*Carex longerostrata* var. *pallida* (Kitag.) Ohwi
Angiospermae	Cyperaceae	*Gahnia tristis* Nees in Hooker & Arnott
Angiospermae	Cyperaceae	*Hypolytrum nemorum* (Vahl) Spreng.
Angiospermae	Cyperaceae	*Rhynchospora rubra* (Lour.) Makino
Angiospermae	Cyperaceae	*Scleria biflora* Roxb.
Angiospermae	Cyperaceae	*Scleria levis* Retz
Angiospermae	Cyperaceae	*Scleria terrestris* (L.) Fassett
Angiospermae	Poaceae	*Cyrtococcum patens* (L.) A. Camus
Angiospermae	Poaceae	*Indocalamus latifolius* (Keng) McClure
Angiospermae	Poaceae	*Indocalamus longiauritus* Hand.-Mazz.
Angiospermae	Poaceae	*Indocalamus tessellatus* (Munro) P. C. Keng
Angiospermae	Poaceae	*Ischaemum ciliare* Retz.
Angiospermae	Poaceae	*Lophatherum gracile* Brongn.
Angiospermae	Poaceae	*Microstegium fasciculatum* (L.) Henrard
Angiospermae	Poaceae	*Miscanthus floridulus* (Labill.) Warburg ex K. Schumann
Angiospermae	Poaceae	*Miscanthus sinensis* Andersson
Angiospermae	Poaceae	*Neyraudia reynaudiana* (Kunth) Keng ex Hitchc.
Angiospermae	Poaceae	*Oplismenus undulatifolius* (Ard.) Roemer & Schuit.
Angiospermae	Poaceae	*Ottochloa nodosa* var. *micrantha* (Balansa ex A. Camus) S. L. Chen & S. M. Phillips
Angiospermae	Poaceae	*Panicum repens* L.
Angiospermae	Poaceae	*Pennisetum purpureum* Schumach.

Taxonomic groups	Family	Species
Angiospermae	Poaceae	*Phragmites australis* (Cav.) Trin. ex Steud.
Angiospermae	Poaceae	*Phyllostachys nidularia* Munro
Angiospermae	Poaceae	*Pleioblastus amarus* (Keng) P. C. Keng
Angiospermae	Poaceae	*Schizostachyum dumetorum* (Hance ex Walp.) Munro
Angiospermae	Poaceae	*Setaria geniculata* (Lam.) Beauv.
Angiospermae	Poaceae	*Thysanolaena latifolia* (Roxb. ex Hornem.) Honda
Angiospermae	Lardizabalaceae	*Holboellia angustifolia* Wall.
Angiospermae	Lardizabalaceae	*Holboellia grandiflora* Réaub.
Angiospermae	Lardizabalaceae	*Stauntonia chinensis* DC.
Angiospermae	Menispermaceae	*Cyclea barbata* Miers
Angiospermae	Menispermaceae	*Cyclea hypoglauca* (Schauer) Diels in Engler
Angiospermae	Menispermaceae	*Cyclea racemosa* Oliv.
Angiospermae	Menispermaceae	*Diploclisia affinis* (Oliv.) Diels in Engler
Angiospermae	Menispermaceae	*Diploclisia glaucescens* (Blume) Diels in Engler
Angiospermae	Menispermaceae	*Hypserpa nitida* Miers
Angiospermae	Menispermaceae	*Pericampylus glaucus* (Lam.) Merr.
Angiospermae	Ranunculaceae	*Clematis crassifolia* Benth.
Angiospermae	Ranunculaceae	*Clematis finetiana* H. Lév. & Vaniot
Angiospermae	Ranunculaceae	*Clematis florida* Thunb.
Angiospermae	Ranunculaceae	*Clematis loureiroana* DC.
Angiospermae	Ranunculaceae	*Clematis uncinata* Champ. & Benth.
Angiospermae	Sabiaceae	*Meliosma rigida* Siebold & Zucc.
Angiospermae	Sabiaceae	*Meliosma squamulata* Hance
Angiospermae	Sabiaceae	*Meliosma thorelii* Lecomte
Angiospermae	Sabiaceae	*Sabia limoniacea* Wall. ex Hook. f. & Thomson
Angiospermae	Proteaceae	*Helicia cochinchinensis* Lour.
Angiospermae	Proteaceae	*Helicia reticulata* W. T. Wang
Angiospermae	Dilleniaceae	*Tetracera sarmentosa* (L.) Vahl
Angiospermae	Altingiaceae	*Altingia chinensis* (Champ. ex Benth.) Oliv. ex Hance
Angiospermae	Altingiaceae	*Liquidambar formosana* Hance
Angiospermae	Hamamelidaceae	*Distyliopsis tutcheri* (Hemsl.) P. K. Endress
Angiospermae	Hamamelidaceae	*Distylium myricoides* Hemsl.
Angiospermae	Hamamelidaceae	*Eustigma oblongifolium* Gardner & Champ.
Angiospermae	Hamamelidaceae	*Loropetalum chinense* (R. Br.) Oliv.
Angiospermae	Daphniphyllaceae	*Daphniphyllum calycinum* Benth.
Angiospermae	Daphniphyllaceae	*Daphniphyllum oldhamii* (Hemsl.) K. Rosenth.
Angiospermae	Iteaceae	*Itea chinensis* Hook. & Arn.
Angiospermae	Vitaceae	*Causonis corniculata* (Benth.) J. Wen & L. M. Lu
Angiospermae	Vitaceae	*Causonis japonica* (Thunb.) Raf.
Angiospermae	Vitaceae	*Nekemias cantoniensis* (Hook. & Arn.) J. Wen & Z. L. Nie
Angiospermae	Vitaceae	*Nekemias grossedentata* (Hand.-Mazz.) J. Wen & Z. L. Nie
Angiospermae	Vitaceae	*Tetrastigma hemsleyanum* Diels & Gilg
Angiospermae	Fabaceae	*Abrus precatorius* L.

Taxonomic groups	Family	Species
Angiospermae	Fabaceae	*Abrus pulchellus* subsp. *mollis* (Hance) Verdc.
Angiospermae	Fabaceae	*Acacia mangium* Willd.
Angiospermae	Fabaceae	*Adenanthera microsperma* Teijsm. & Binn.
Angiospermae	Fabaceae	*Albizia corniculata* (Lour.) Druce
Angiospermae	Fabaceae	*Archidendron clypearia* (Jack) I. C. Nielsen
Angiospermae	Fabaceae	*Archidendron lucidum* (Benth.) I. C. Nielsen
Angiospermae	Fabaceae	*Archidendron utile* (Chun & F. C. How) I. C. Nielsen
Angiospermae	Fabaceae	*Bauhinia purpurea* L.
Angiospermae	Fabaceae	*Biancaea millettii* (Hook. & Arn.) Gagnon & G. P. Lewis
Angiospermae	Fabaceae	*Bowringia callicarpa* Champ. ex Benth.
Angiospermae	Fabaceae	*Caesalpinia crista* L.
Angiospermae	Fabaceae	*Caesalpinia vernalis* Champ.
Angiospermae	Fabaceae	*Callerya dielsiana* (Harms) P. K. Lôc ex Z. Wei & Pedley
Angiospermae	Fabaceae	*Callerya nitida* (Benth.) R. Geesink
Angiospermae	Fabaceae	*Cheniella glauca* (Benth.) R. Clark & Mackinder
Angiospermae	Fabaceae	*Dalbergia benthamii* Prain
Angiospermae	Fabaceae	*Dalbergia hancei* Benth.
Angiospermae	Fabaceae	*Dalbergia hupeana* Hance
Angiospermae	Fabaceae	*Dalbergia millettii* Benth.
Angiospermae	Fabaceae	*Gleditsia fera* (Lour.) Merr.
Angiospermae	Fabaceae	*Mucuna sempervirens* Hemsl.
Angiospermae	Fabaceae	*Nanhaia speciosa* (Champ. ex Benth.) J. Compton & Schrire
Angiospermae	Fabaceae	*Ormosia indurata* H. Y. Chen
Angiospermae	Fabaceae	*Ormosia semicastrata* Hance
Angiospermae	Fabaceae	*Phanera championii* Benth.
Angiospermae	Fabaceae	*Pueraria montana* (Lour.) Merr.
Angiospermae	Fabaceae	*Wisteriopsis championii* (Benth.) J. Compton & Schrire
Angiospermae	Fabaceae	*Wisteriopsis reticulata* (Benth.) J. Compton & Schrire
Angiospermae	Polygalaceae	*Polygala fallax* Hemsl.
Angiospermae	Rosaceae	*Eriobotrya cavaleriei* (H. Lév.) Rehder
Angiospermae	Rosaceae	*Eriobotrya fragrans* Champ. ex Benth.
Angiospermae	Rosaceae	*Photinia glabra* (Thunb.) Maxim.
Angiospermae	Rosaceae	*Photinia prunifolia* (Hook. & Arn.) Lindl.
Angiospermae	Rosaceae	*Photinia raupingensis* K. C. Kuan
Angiospermae	Rosaceae	*Prunus phaeosticta* (Hance) Maxim.
Angiospermae	Rosaceae	*Pygeum topengii* Merr.
Angiospermae	Rosaceae	*Rhaphiolepis indica* (L.) Lindl.
Angiospermae	Rosaceae	*Rosa laevigata* Michx.
Angiospermae	Rosaceae	*Rubus alceifolius* Poir.
Angiospermae	Rosaceae	*Rubus corchorifolius* L. f.
Angiospermae	Rosaceae	*Rubus leucanthus* Hance
Angiospermae	Rosaceae	*Rubus reflexus* Ker Gawl.
Angiospermae	Rhamnaceae	*Berchemia floribunda* (Wall.) Brongn.

Taxonomic groups	Family	Species
Angiospermae	Rhamnaceae	*Sageretia lucida* Merr.
Angiospermae	Rhamnaceae	*Sageretia thea* (Osbeck) M. C. Johnst.
Angiospermae	Rhamnaceae	*Ventilago leiocarpa* Benth.
Angiospermae	Cannabaceae	*Celtis sinensis* Pers.
Angiospermae	Cannabaceae	*Celtis timorensis* Span.
Angiospermae	Cannabaceae	*Trema tomentosa* (Roxb.) H. Hara
Angiospermae	Moraceae	*Artocarpus hypargyreus* Hance
Angiospermae	Moraceae	*Ficus erecta* Thunb.
Angiospermae	Moraceae	*Ficus fistulosa* Reinw. ex Blume
Angiospermae	Moraceae	*Ficus formosana* Maxim.
Angiospermae	Moraceae	*Ficus hirta* Vahl
Angiospermae	Moraceae	*Ficus hispida* L. f.
Angiospermae	Moraceae	*Ficus microcarpa* L. f.
Angiospermae	Moraceae	*Ficus nervosa* B. Heyne ex Roth
Angiospermae	Moraceae	*Ficus pumila* L.
Angiospermae	Moraceae	*Ficus pyriformis* Hook. & Arn.
Angiospermae	Moraceae	*Ficus sarmentosa* var. *impressa* (Champ.) Corner
Angiospermae	Moraceae	*Ficus subpisocarpa* Gagnep.
Angiospermae	Moraceae	*Ficus variegata* Blume
Angiospermae	Moraceae	*Ficus variolosa* Lindl. ex Benth.
Angiospermae	Moraceae	*Ficus vasculosa* Wall. ex Miq.
Angiospermae	Moraceae	*Maclura cochinchinensis* (Lour.) Corner
Angiospermae	Moraceae	*Malaisia scandens* (Lour.) Planch.
Angiospermae	Urticaceae	*Boehmeria nivea* (L.) Gaudich.
Angiospermae	Urticaceae	*Pellionia radicans* (Siebold & Zucc.) Wedd.
Angiospermae	Urticaceae	*Pellionia scabra* Benth.
Angiospermae	Fagaceae	*Castanopsis carlesii* (Hemsl.) Hayata
Angiospermae	Fagaceae	*Castanopsis eyrei* (Champ. ex Benth.) Tutcher
Angiospermae	Fagaceae	*Castanopsis fabri* Hance
Angiospermae	Fagaceae	*Castanopsis fargesii* Franch.
Angiospermae	Fagaceae	*Castanopsis fissa* (Champ. ex Benth.) Rehder & E. H. Wilson in Sarg.
Angiospermae	Fagaceae	*Castanopsis lamontii* Hance
Angiospermae	Fagaceae	*Lithocarpus corneus* (Lour.) Rehder
Angiospermae	Fagaceae	*Lithocarpus hancei* (Benth.) Rehder
Angiospermae	Fagaceae	*Lithocarpus uvariifolius* (Hance) Rehder
Angiospermae	Fagaceae	*Quercus championii* Benth.
Angiospermae	Fagaceae	*Quercus chungii* F. P. Metcalf
Angiospermae	Fagaceae	*Quercus hui* Chun
Angiospermae	Fagaceae	*Quercus myrsinifolia* Blume
Angiospermae	Fagaceae	*Quercus neglecta* (Schottky) Koidz.
Angiospermae	Myricaceae	*Morella rubra* Lour.
Angiospermae	Juglandaceae	*Engelhardia roxburghiana* Wall.
Angiospermae	Cucurbitaceae	*Gynostemma pentaphyllum* (Thunb.) Makino

Taxonomic groups	Family	Species
Angiospermae	Cucurbitaceae	*Thladiantha cordifolia* (Blume) Cogn.
Angiospermae	Begoniaceae	*Begonia palmata* var. *bowringiana* (Champ. ex Benth.) Golding & Kareg.
Angiospermae	Celastraceae	*Celastrus hindsii* Benth.
Angiospermae	Celastraceae	*Celastrus monospermus* Roxb.
Angiospermae	Celastraceae	*Celastrus orbiculatus* Thunb.
Angiospermae	Celastraceae	*Euonymus fortunei* (Turcz.) Hand.-Mazz.
Angiospermae	Celastraceae	*Euonymus laxiflorus* Champ. & Benth.
Angiospermae	Celastraceae	*Euonymus nitidus* Benth.
Angiospermae	Celastraceae	*Loeseneriella concinna* A. C. Sm.
Angiospermae	Celastraceae	*Microtropis reticulata* Dunn
Angiospermae	Connaraceae	*Connarus paniculatus* Roxb.
Angiospermae	Connaraceae	*Rourea microphylla* (Hook. & Arn.) Planch.
Angiospermae	Connaraceae	*Rourea minor* (Gaertn.) Leenh.
Angiospermae	Elaeocarpaceae	*Elaeocarpus chinensis* (Gardner & Champ.) Hook. f. ex Benth.
Angiospermae	Elaeocarpaceae	*Elaeocarpus decipiens* Hemsl.
Angiospermae	Elaeocarpaceae	*Elaeocarpus glabripetalus* Merr.
Angiospermae	Elaeocarpaceae	*Elaeocarpus japonicus* Siebold & Zucc.
Angiospermae	Elaeocarpaceae	*Elaeocarpus sylvestris* (Lour.) Poir.
Angiospermae	Elaeocarpaceae	*Sloanea sinensis* (Hance) Hemsl.
Angiospermae	Rhizophoraceae	*Carallia brachiata* (Lour.) Merr.
Angiospermae	Clusiaceae	*Garcinia multiflora* Champ. ex Benth.
Angiospermae	Clusiaceae	*Garcinia oblongifolia* Champ. ex Benth.
Angiospermae	Calophyllaceae	*Calophyllum membranaceum* Gardner & Champ.
Angiospermae	Hypericaceae	*Cratoxylum cochinchinense* (Lour.) Blume
Angiospermae	Violaceae	*Viola diffusa* Ging. in DC.
Angiospermae	Salicaceae	*Casearia glomerata* Roxb. ex DC.
Angiospermae	Salicaceae	*Casearia membranacea* Hance
Angiospermae	Salicaceae	*Casearia velutina* Blume
Angiospermae	Salicaceae	*Homalium cochinchinense* (Lour.) Druce
Angiospermae	Salicaceae	*Idesia polycarpa* Maxim.
Angiospermae	Salicaceae	*Scolopia chinensis* (Lour.) Clos
Angiospermae	Salicaceae	*Scolopia saeva* (Hance) Hance
Angiospermae	Salicaceae	*Xylosma congesta* (Lour.) Merr.
Angiospermae	Salicaceae	*Xylosma longifolia* Clos
Angiospermae	Euphorbiaceae	*Croton lachynocarpus* Benth.
Angiospermae	Euphorbiaceae	*Endospermum chinense* Benth.
Angiospermae	Euphorbiaceae	*Hancea hookeriana* Seem.
Angiospermae	Euphorbiaceae	*Macaranga sampsonii* Hance
Angiospermae	Euphorbiaceae	*Macaranga tanarius* var. *tomentosa* (Blume) Müll. Arg.
Angiospermae	Euphorbiaceae	*Mallotus apelta* (Lour.) Müll. Arg.
Angiospermae	Euphorbiaceae	*Mallotus paniculatus* (Lam.) Müll. Arg.
Angiospermae	Euphorbiaceae	*Mallotus philippensis* (Lamarck) Müll. Arg.
Angiospermae	Euphorbiaceae	*Mallotus tenuifolius* Pax

Taxonomic groups	Family	Species
Angiospermae	Euphorbiaceae	*Triadica cochinchinensis* Lour.
Angiospermae	Euphorbiaceae	*Triadica sebifera* (L.) Small
Angiospermae	Ixonanthaceae	*Ixonanthes reticulata* Jack
Angiospermae	Phyllanthaceae	*Antidesma bunius* (L.) Spreng.
Angiospermae	Phyllanthaceae	*Antidesma fordii* Hemsl.
Angiospermae	Phyllanthaceae	*Antidesma japonicum* Siebold & Zucc.
Angiospermae	Phyllanthaceae	*Antidesma montanum* Blume
Angiospermae	Phyllanthaceae	*Antidesma montanum* var. *microphyllum* (Hemsl.) Petra Hoffm.
Angiospermae	Phyllanthaceae	*Aporosa dioica* (Roxb.) Müll. Arg.
Angiospermae	Phyllanthaceae	*Bischofia javanica* Blume
Angiospermae	Phyllanthaceae	*Breynia fruticosa* (L.) Hook. f.
Angiospermae	Phyllanthaceae	*Bridelia balansae* Tutcher
Angiospermae	Phyllanthaceae	*Bridelia tomentosa* Blume
Angiospermae	Phyllanthaceae	*Glochidion eriocarpum* Champ. ex Benth.
Angiospermae	Phyllanthaceae	*Glochidion hirsutum* (Roxb.) Voigt
Angiospermae	Phyllanthaceae	*Glochidion wrightii* Benth.
Angiospermae	Phyllanthaceae	*Glochidion zeylanicum* (Gaertn.) A. Juss.
Angiospermae	Phyllanthaceae	*Phyllanthus cochinchinensis* (Lour.) Spreng.
Angiospermae	Phyllanthaceae	*Phyllanthus emblica* L.
Angiospermae	Phyllanthaceae	*Phyllanthus reticulatus* Poir. in Lamark
Angiospermae	Phyllanthaceae	*Phyllanthus urinaria* L.
Angiospermae	Myrtaceae	*Baeckea frutescens* L.
Angiospermae	Myrtaceae	*Rhodomyrtus tomentosa* (Aiton) Hassk.
Angiospermae	Myrtaceae	*Syzygium buxifolium* Hook. & Arn.
Angiospermae	Myrtaceae	*Syzygium championii* (Benth.) Merr. & L. M. Perry
Angiospermae	Myrtaceae	*Syzygium hancei* Merr. & L. M. Perry
Angiospermae	Myrtaceae	*Syzygium jambos* (L.) Alston
Angiospermae	Myrtaceae	*Syzygium levinei* (Merr.) Merr. & L. M. Perry
Angiospermae	Myrtaceae	*Syzygium nervosum* DC.
Angiospermae	Myrtaceae	*Syzygium rehderianum* Merr. & L. M. Perry
Angiospermae	Melastomataceae	*Barthea barthei* (Hance) Krass.
Angiospermae	Melastomataceae	*Blastus cochinchinensis* Lour.
Angiospermae	Melastomataceae	*Blastus pauciflorus* (Benth.) Guillaumin
Angiospermae	Melastomataceae	*Melastoma candidum* D. Don
Angiospermae	Melastomataceae	*Melastoma dodecandrum* Lour.
Angiospermae	Melastomataceae	*Melastoma malabathricum* L.
Angiospermae	Melastomataceae	*Melastoma normale* D. Don
Angiospermae	Melastomataceae	*Melastoma sanguineum* Sims
Angiospermae	Melastomataceae	*Memecylon ligustrifolium* Champ.
Angiospermae	Melastomataceae	*Memecylon nigrescens* Hook. & Arn.
Angiospermae	Staphyleaceae	*Euscaphis japonica* (Thunb. ex Roem. & Schult.) Kanitz
Angiospermae	Staphyleaceae	*Turpinia arguta* (Lindl.) Seem.
Angiospermae	Staphyleaceae	*Turpinia montana* (Blume) Kurz.

Taxonomic groups	Family	Species
Angiospermae	Burseraceae	*Canarium subulatum* Guillaumin
Angiospermae	Anacardiaceae	*Choerospondias axillaris* (Roxb.) B. L. Burtt & A. W. Hill
Angiospermae	Anacardiaceae	*Mangifera indica* L.
Angiospermae	Anacardiaceae	*Rhus chinensis* Mill.
Angiospermae	Anacardiaceae	*Toxicodendron succedaneum* (L.) Kuntze
Angiospermae	Anacardiaceae	*Toxicodendron vernicifluum* (Stokes) F. A. Barkley
Angiospermae	Sapindaceae	*Acer fabri* Hance
Angiospermae	Sapindaceae	*Acer lucidum* F. P. Metcalf
Angiospermae	Sapindaceae	*Acer sino-oblongum* F. P. Metcalf
Angiospermae	Sapindaceae	*Acer tutcheri* Duthie
Angiospermae	Sapindaceae	*Dimocarpus longan* Lour.
Angiospermae	Sapindaceae	*Litchi chinensis* Sonn.
Angiospermae	Rutaceae	*Acronychia pedunculata* (L.) Miq.
Angiospermae	Rutaceae	*Atalantia buxifolia* (Poir.) Oliv.
Angiospermae	Rutaceae	*Citrus japonica* Thunb.
Angiospermae	Rutaceae	*Glycosmis parviflora* Kurz.
Angiospermae	Rutaceae	*Melicope pteleifolia* (Champ. ex Benth.) Hartley
Angiospermae	Rutaceae	*Tetradium glabrifolium* (Champ. ex Benth.) T. G. Hartley
Angiospermae	Rutaceae	*Toddalia asiatica* (L.) Lam.
Angiospermae	Rutaceae	*Zanthoxylum austrosinense* C. C. Huang
Angiospermae	Rutaceae	*Zanthoxylum avicennae* (Lam.) DC.
Angiospermae	Rutaceae	*Zanthoxylum nitidum* (Roxb.) DC.
Angiospermae	Rutaceae	*Zanthoxylum scandens* Blume
Angiospermae	Simaroubaceae	*Brucea javanica* (L.) Merr.
Angiospermae	Malvaceae	*Byttneria grandifolia* DC.
Angiospermae	Malvaceae	*Helicteres angustifolia* L.
Angiospermae	Malvaceae	*Microcos paniculata* L.
Angiospermae	Malvaceae	*Reevesia thyrsoidea* Lindl.
Angiospermae	Malvaceae	*Sterculia lanceolata* Cav.
Angiospermae	Thymelaeaceae	*Aquilaria sinensis* (Lour.) Spreng.
Angiospermae	Thymelaeaceae	*Wikstroemia indica* (L.) C. A. Mey.
Angiospermae	Thymelaeaceae	*Wikstroemia nutans* Champ. ex Benth.
Angiospermae	Capparaceae	*Capparis acutifolia* Sweet
Angiospermae	Capparaceae	*Capparis cantoniensis* Lour.
Angiospermae	Balanophoraceae	*Balanophora harlandii* Hook. f.
Angiospermae	Santalaceae	*Dendrotrophe varians* (Blume) Miq.
Angiospermae	Santalaceae	*Pyrularia edulis* (Wall.) A. DC.
Angiospermae	Schoepfiaceae	*Schoepfia chinensis* Gardner & Champ.
Angiospermae	Schoepfiaceae	*Schoepfia jasminodora* Siebold & Zucc.
Angiospermae	Loranthaceae	*Taxillus chinensis* (DC.) Danser
Angiospermae	Cornaceae	*Alangium chinense* (Lour.) Harms
Angiospermae	Cornaceae	*Alangium kurzii* Craib
Angiospermae	Pentaphylacaceae	*Adinandra millettii* (Hook. & Arn.) Benth. & Hook. f. ex Hance

**Taxonomic groups**	**Family**	**Species**
Angiospermae	Pentaphylacaceae	*Eurya acutisepala* Hu & L. K. Ling
Angiospermae	Pentaphylacaceae	*Eurya chinensis* R. Brown in C. Abel
Angiospermae	Pentaphylacaceae	*Eurya ciliata* Merr.
Angiospermae	Pentaphylacaceae	*Eurya distichophylla* Hemsl.
Angiospermae	Pentaphylacaceae	*Eurya hebeclados* Ling
Angiospermae	Pentaphylacaceae	*Eurya loquaiana* Dunn
Angiospermae	Pentaphylacaceae	*Eurya macartneyi* Champion
Angiospermae	Pentaphylacaceae	*Eurya muricata* Dunn
Angiospermae	Pentaphylacaceae	*Eurya nitida* Korth.
Angiospermae	Pentaphylacaceae	*Pentaphylax euryoides* Gardner & Champ.
Angiospermae	Pentaphylacaceae	*Ternstroemia gymnanthera* (Wight & Arn.) Bedd.
Angiospermae	Sapotaceae	*Sarcosperma laurinum* (Benth.) Hook. f.
Angiospermae	Sapotaceae	*Sinosideroxylon pedunculatum* (Hemsl.) H. Chuang
Angiospermae	Sapotaceae	*Sinosideroxylon wightianum* (Hook. & Arn.) Aubrév.
Angiospermae	Ebenaceae	*Diospyros chunii* F. P. Metcalf & L. Chen
Angiospermae	Ebenaceae	*Diospyros eriantha* Champion ex Bentham
Angiospermae	Ebenaceae	*Diospyros morrisiana* Hance
Angiospermae	Ebenaceae	*Diospyros strigosa* Hemsl.
Angiospermae	Ebenaceae	*Diospyros vaccinioides* Lindl.
Angiospermae	Primulaceae	*Ardisia crenata* Sims
Angiospermae	Primulaceae	*Ardisia crispa* (Thunb.) A. DC.
Angiospermae	Primulaceae	*Ardisia hanceana* Mez
Angiospermae	Primulaceae	*Ardisia lindleyana* D. Dietr.
Angiospermae	Primulaceae	*Ardisia mamillata* Hance
Angiospermae	Primulaceae	*Ardisia primulifolia* Gardner & Champ.
Angiospermae	Primulaceae	*Ardisia quinquegona* Blume
Angiospermae	Primulaceae	*Embelia laeta* (L.) Mez
Angiospermae	Primulaceae	*Embelia ribes* Burm. f.
Angiospermae	Primulaceae	*Embelia vestita* Roxb.
Angiospermae	Primulaceae	*Maesa japonica* (Thunb.) Moritzi
Angiospermae	Primulaceae	*Maesa perlarius* (Lour.) Merr.
Angiospermae	Primulaceae	*Maesa salicifolia* E. Walker
Angiospermae	Primulaceae	*Myrsine linearis* (Lour.) Poir.
Angiospermae	Primulaceae	*Myrsine seguinii* H. Lév.
Angiospermae	Theaceae	*Camellia caudata* Wall.
Angiospermae	Theaceae	*Camellia granthamiana* Sealy
Angiospermae	Theaceae	*Camellia kissi* Wall.
Angiospermae	Theaceae	*Camellia salicifolia* Champion ex Bentham
Angiospermae	Theaceae	*Polyspora axillaris* (Roxb. ex Ker Gawl.) Sweet
Angiospermae	Theaceae	*Pyrenaria microcarpa* Keng
Angiospermae	Theaceae	*Pyrenaria spectabilis* (Champ.) C. Y. Wu & S. X. Yang ex S. X. Yang
Angiospermae	Theaceae	*Schima superba* Gardner & Champ.
Angiospermae	Symplocaceae	*Symplocos adenophylla* Wall.

Taxonomic groups	Family	Species
Angiospermae	Symplocaceae	*Symplocos chinensis* (Lour.) Druce
Angiospermae	Symplocaceae	*Symplocos congesta* Benth.
Angiospermae	Symplocaceae	*Symplocos crassilimba* Merr.
Angiospermae	Symplocaceae	*Symplocos glauca* (Thunb.) Koidz.
Angiospermae	Symplocaceae	*Symplocos lancifolia* Siebold & Zucc.
Angiospermae	Symplocaceae	*Symplocos lucida* (Thunb.) Siebold & Zucc.
Angiospermae	Symplocaceae	*Symplocos phyllocalyx* Clarke
Angiospermae	Symplocaceae	*Symplocos stellaris* Brand
Angiospermae	Symplocaceae	*Symplocos sumuntia* Buch.-Ham. ex D. Don
Angiospermae	Symplocaceae	*Symplocos tanakana* Nakai
Angiospermae	Symplocaceae	*Symplocos theophrastifolia* Siebold & Zucc.
Angiospermae	Styracaceae	*Huodendron biaristatum* var. *parviflorum* (Merr.) Rehder
Angiospermae	Styracaceae	*Rehderodendron kwangtungense* Chun
Angiospermae	Styracaceae	*Styrax agrestis* (Lour.) G. Don
Angiospermae	Styracaceae	*Styrax odoratissimus* Champion ex Bentham
Angiospermae	Styracaceae	*Styrax suberifolius* Hook. & Arn.
Angiospermae	Actinidiaceae	*Actinidia latifolia* (Gardner & Champ.) Merr.
Angiospermae	Actinidiaceae	*Saurauia tristyla* DC.
Angiospermae	Ericaceae	*Enkianthus quinqueflorus* Lour.
Angiospermae	Ericaceae	*Rhododendron farrerae* Sweet
Angiospermae	Ericaceae	*Rhododendron moulmainense* Hook.
Angiospermae	Ericaceae	*Rhododendron simsii* Planch.
Angiospermae	Ericaceae	*Vaccinium bracteatum* Thunb.
Angiospermae	Ericaceae	*Vaccinium carlesii* Dunn
Angiospermae	Icacinaceae	*Mappianthus iodoides* Hand.-Mazz.
Angiospermae	Garryaceae	*Aucuba chinensis* Benth.
Angiospermae	Rubiaceae	*Adina pilulifera* (Lam.) Franch. ex Drake
Angiospermae	Rubiaceae	*Aidia canthioides* (Champ. ex Benth.) Masam.
Angiospermae	Rubiaceae	*Aidia cochinchinensis* Lour.
Angiospermae	Rubiaceae	*Alleizettella leucocarpa* (Champ. ex Benth.) Tirveng.
Angiospermae	Rubiaceae	*Antirhea chinensis* (Champ. ex Benth.) Benth. & Hook. f. ex F. B. Forbes & Hemsl.
Angiospermae	Rubiaceae	*Coptosapelta diffusa* (Champ. ex Benth.) Steenis
Angiospermae	Rubiaceae	*Diplospora dubia* (Lindl.) Masam.
Angiospermae	Rubiaceae	*Gardenia jasminoides* J. Ellis
Angiospermae	Rubiaceae	*Hedyotis acutangula* Champ. ex Benth.
Angiospermae	Rubiaceae	*Hedyotis auricularia* L.
Angiospermae	Rubiaceae	*Hedyotis caudatifolia* Merr. & F. P. Metcalf
Angiospermae	Rubiaceae	*Hedyotis hedyotidea* (DC.) Merr.
Angiospermae	Rubiaceae	*Hedyotis loganioides* Benth.
Angiospermae	Rubiaceae	*Lasianthus chinensis* (Champ. ex Benth.) Benth.
Angiospermae	Rubiaceae	*Lasianthus japonicus* Miq.
Angiospermae	Rubiaceae	*Metadina trichotoma* (Zoll. & Moritzi) Bakh. f.

**Taxonomic groups**	**Family**	**Species**
Angiospermae	Rubiaceae	*Morinda parvifolia* Bartl. ex DC.
Angiospermae	Rubiaceae	*Morinda umbellata* subsp. *obovata* Y. Z. Ruan
Angiospermae	Rubiaceae	*Mussaenda kwangtungensis* H. L. Li
Angiospermae	Rubiaceae	*Mussaenda pubescens* W. T. Aiton
Angiospermae	Rubiaceae	*Ophiorrhiza brevidentata* H. S. Lo
Angiospermae	Rubiaceae	*Ophiorrhiza cantonensis* Hance
Angiospermae	Rubiaceae	*Ophiorrhiza japonica* Blume
Angiospermae	Rubiaceae	*Ophiorrhiza pumila* Champ. & Benth.
Angiospermae	Rubiaceae	*Paederia foetida* L.
Angiospermae	Rubiaceae	*Pavetta hongkongensis* Bremek.
Angiospermae	Rubiaceae	*Psychotria asiatica* L.
Angiospermae	Rubiaceae	*Psychotria serpens* L.
Angiospermae	Rubiaceae	*Psydrax dicocca* Gaertn.
Angiospermae	Rubiaceae	*Tarenna attenuata* (Hook. f.) Hutch. in Sargent
Angiospermae	Rubiaceae	*Tarenna mollissima* (Hook. & Arn.) B. L. Rob.
Angiospermae	Gentianaceae	*Tripterospermum chinense* (Migo) Harry Sm.
Angiospermae	Loganiaceae	*Gardneria multiflora* Makino
Angiospermae	Loganiaceae	*Strychnos cathayensis* Merr.
Angiospermae	Gelsemiaceae	*Gelsemium elegans* (Gardner & Champ.) Benth.
Angiospermae	Apocynaceae	*Alyxia sinensis* Champ. ex Benth.
Angiospermae	Apocynaceae	*Cerbera manghas* L.
Angiospermae	Apocynaceae	*Cynanchum auriculatum* Royle ex Wight
Angiospermae	Apocynaceae	*Gymnema sylvestre* (Retz.) R. Br. ex Schult. in Roem. & Schult.
Angiospermae	Apocynaceae	*Melodinus cochinchinensis* (Lour.) Merr.
Angiospermae	Apocynaceae	*Melodinus fusiformis* Champ. ex Benth.
Angiospermae	Apocynaceae	*Strophanthus divaricatus* (Lour.) Hook. & Arn.
Angiospermae	Apocynaceae	*Toxocarpus wightianus* Hook. & Arn.
Angiospermae	Apocynaceae	*Trachelospermum jasminoides* (Lindl.) Lem.
Angiospermae	Apocynaceae	*Tylophora ovata* (Lindl.) Hook. ex Steud.
Angiospermae	Apocynaceae	*Urceola rosea* (Hook. & Arn.) D. J. Middleton
Angiospermae	Boraginaceae	*Ehretia longiflora* Champ. ex Benth.
Angiospermae	Oleaceae	*Chengiodendron matsumuranum* (Hayata) C. B. Shang, X. R. Wang, Yi F. Duan & Yong F. Li
Angiospermae	Oleaceae	*Fraxinus insularis* Hemsl.
Angiospermae	Oleaceae	*Jasminum lanceolaria* Roxb.
Angiospermae	Oleaceae	*Ligustrum lianum* Hsu
Angiospermae	Oleaceae	*Ligustrum sinense* Lour.
Angiospermae	Oleaceae	*Osmanthus cooperi* Hemsl.
Angiospermae	Gesneriaceae	*Oreocharis benthamii* C. B. Clarke
Angiospermae	Gesneriaceae	*Oreocharis maximowiczii* C. B. Clarke
Angiospermae	Gesneriaceae	*Primulina dryas* (Dunn) Mich. Möller & A. Weber
Angiospermae	Acanthaceae	*Dicliptera chinensis* (L.) Juss.
Angiospermae	Acanthaceae	*Justicia championii* T. Anderson

Taxonomic groups	Family	Species
Angiospermae	Acanthaceae	*Justicia quadrifaria* (Nees) T. Anderson
Angiospermae	Acanthaceae	*Strobilanthes oliganthus* Miq.
Angiospermae	Lamiaceae	*Callicarpa bodinieri* H. Lév.
Angiospermae	Lamiaceae	*Callicarpa giraldii* Hesse ex Rehder
Angiospermae	Lamiaceae	*Callicarpa integerrima* Champ.
Angiospermae	Lamiaceae	*Callicarpa kochiana* Makino
Angiospermae	Lamiaceae	*Callicarpa kwangtungensis* Chun
Angiospermae	Lamiaceae	*Callicarpa pedunculata* R. Br.
Angiospermae	Lamiaceae	*Callicarpa rubella* Lindl.
Angiospermae	Lamiaceae	*Clerodendrum cyrtophyllum* Turcz.
Angiospermae	Lamiaceae	*Clerodendrum fortunatum* L.
Angiospermae	Lamiaceae	*Clinopodium polycephalum* (Vaniot) C. Y. Wu & S. J. Hsuan ex P. S. Hsu
Angiospermae	Lamiaceae	*Vitex quinata* (Lour.) Will.
Angiospermae	Aquifoliaceae	*Ilex asprella* (Hook. & Arn.) Champ. ex Benth.
Angiospermae	Aquifoliaceae	*Ilex confertiflora* Merr.
Angiospermae	Aquifoliaceae	*Ilex ficoidea* Hemsl.
Angiospermae	Aquifoliaceae	*Ilex graciliflora* Champ.
Angiospermae	Aquifoliaceae	*Ilex macrocarpa* Oliv.
Angiospermae	Aquifoliaceae	*Ilex memecylifolia* Champ. ex Benth.
Angiospermae	Aquifoliaceae	*Ilex nitidissima* C. J. Tseng
Angiospermae	Aquifoliaceae	*Ilex pubescens* Hook. & Arn.
Angiospermae	Aquifoliaceae	*Ilex rotunda* Thunb.
Angiospermae	Aquifoliaceae	*Ilex triflora* Blume
Angiospermae	Aquifoliaceae	*Ilex viridis* Champ. ex Benth.
Angiospermae	Asteraceae	*Ainsliaea kawakamii* Hayata
Angiospermae	Asteraceae	*Aster baccharoides* (Benth.) Steetz
Angiospermae	Asteraceae	*Aster indicus* L.
Angiospermae	Asteraceae	*Blumea megacephala* (Randeria) C. C. Chang & Y. Q. Tseng in Y. Ling
Angiospermae	Asteraceae	*Decaneuropsis cumingiana* (Benth.) H. Rob. & Skvarla
Angiospermae	Adoxaceae	*Viburnum odoratissimum* Ker Gawl.
Angiospermae	Adoxaceae	*Viburnum sempervirens* K. Koch
Angiospermae	Caprifoliaceae	*Lonicera acuminata* Wall.
Angiospermae	Caprifoliaceae	*Lonicera confusa* (Sweet) DC.
Angiospermae	Caprifoliaceae	*Lonicera japonica* Thunb.
Angiospermae	Pittosporaceae	*Pittosporum glabratum* Lindl.
Angiospermae	Pittosporaceae	*Pittosporum illicioides* Makino
Angiospermae	Pittosporaceae	*Pittosporum pauciflorum* Hook. & Arn.
Angiospermae	Araliaceae	*Dendropanax dentiger* (Harms) Merr.
Angiospermae	Araliaceae	*Dendropanax proteus* (Champ.) Benth.
Angiospermae	Araliaceae	*Heptapleurum arboricola* Hayata
Angiospermae	Araliaceae	*Heptapleurum heptaphyllum* (L.) Y. F. Deng

Note: The classification systems used are: Pteridophytes-PPG I system, Gymnosperms-GPG I system, Angiosperms-APG IV system.
